# Subluminal Focal Lesions in Peyer's Patches in the Terminal Ileum of Pigs Fed With Different Physical Forms of One Same Diet

**DOI:** 10.3389/fvets.2020.00207

**Published:** 2020-05-15

**Authors:** Maria Grazia Cappai, Corrado Dimauro, Michael Arlinghaus, Saara J. Sander, Walter Pinna, Josef Kamphues

**Affiliations:** ^1^Department of Veterinary Medicine, University of Sassari, Sassari, Italy; ^2^Department of Agriculture, Research Unit of Animal Breeding Sciences, University of Sassari, Sassari, Italy; ^3^Institute of Animal Nutrition, University of Veterinary Medicine Hannover, Hanover, Germany

**Keywords:** diet, grinding intensity, gut, immune homeostasis, particle size, pig

## Abstract

Retrograde backflow of cecum chyme and consequent ascendent colonization of the foregut may occur via the ileocecal valve (IV) under predisposing circumstances. The Peyer's patches (PPs) in the terminal ileum (TI) play a crucial role in targeting antigens and act as a first line of blockage of pathogens in the small intestine. In view of the established impact of the physical form of the diet (grinding and compaction of ingredients) on the physicochemical and microbiological composition of *digesta* throughout the different gastrointestinal tracts, special attention was paid to PP reaction following different dietary treatments. The aim of this study was to explore the effect of different physical forms of one diet (identical for botanical and chemical composition) administered to growing pigs on macro- and microscopic morphology of PPs in the last 3 cm of the TI, as a region of interest immediately close to the IV involved in the prevention of retrograde contamination of the small intestine. The diet effect was tested after 4 weeks of experimental feeding on PPs of 32 growing pigs, fed with four dietary treatments differing for the physical form: FP—finely ground pelleted diet (dMEAN, 0.463 mm); CM—coarsely ground meal diet (dMEAN, 0.880 mm); CP—coarsely ground pelleted diet (dMEAN, 0.836); and CE—coarsely ground extruded diet (dMEAN, 0.659). A higher prevalence of subluminal focal liquefactive necrosis (FLN) in the last 3 cm of the TI was observed in pigs fed with the CE and the FP diet (n. 3/8 or 37.5% and n. = 1/8 or 12.5%, respectively) (*p* = 0.076). FLN negatively and significantly correlated with the pH value of *digesta* of the last part of the small intestine (ρ = −0.361; *p* = 0.026). All animals enrolled appeared clinically healthy throughout the trial. Growth performance were not affected by the different dietary treatments, but fecal dry matter and pH values were affected in a significant way. Results about the morphology of PPs assessed in this trial can be suggestive of the physical form of the diet as a contributing factor to the onset of different antigenic potentials of the intestinal chyme.

## Introduction

In the last decades, pioneer authors (1–4) reported the effects of grinding intensity of cereals alone or in combination with organic acids in the diet of pigs on the reduction of *Salmonella* **spp**. and *Escherichia coli* infections (faster elimination), while favoring the growth of gram-positive bacterial strains, such as lactobacilli and gram-positive cocci in the luminal content of the small intestine of pigs. By contrast, dietetic errors have appeared to be responsible for inducing undesirable characteristics of gut content, including microbial population imbalances toward which the host barriers, such as gastric secretions and intestinal clearance, could fail. Such conditions seem to be involved in the onset of the so-called small intestine bacterial overgrowth (known in human medicine with the acronym SIBO). Neglecting other causes predisposing to SIBO (hypomotility of intestine, for instance) reviewed by Ford et al. (5), inappropriate physicochemical characteristics of the chyme, as a result of the diet, may play as predisposing factors to the colonization of ascending microbial populations from the hindgut to the small intestine.

**Figure 1:**
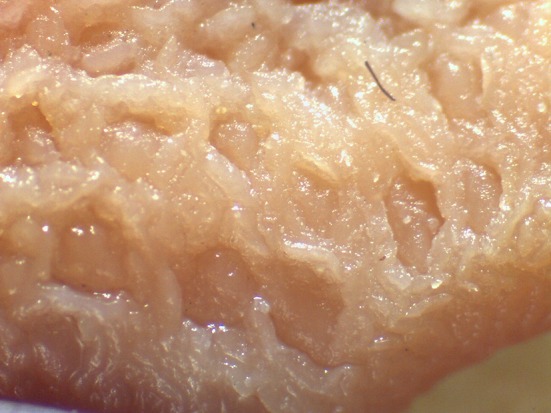
Appearance of the Peyer's patch in the last 3 cm of the terminal ileum of a pig after 48 h of fixation in glutaraldehyde 2.5% (v/v). Particular of the mucosa at stereomicroscope 24×.

Beyond the possibility to modulate the gut microbial composition through feed processing, the integrity of the mucosal barrier of different gastrointestinal tracts (GITs) as well as the composition of *digesta* were reported to be affected by the physical form of the diet (1, 2, 6–13). In particular, coliform proportions in the small intestine can increase in spite of lactobacilli when extremely ground and processed diets are fed to growing pigs (8). In addition, effects of the physical form of the diet on crypt depth and villus height in the small intestine (1, 14) were reported. Finally, the terminal ileum (TI) and the ileocecal valve (IV) were recently described to vary according to the physical form of the diet fed to pigs (15). In particular, the thickness of the sphincter of the IV can be apparently modified according to the type of processing of ingredients, potentially leading to different extents of efficacious prevention of chyme backflow. In light of such circumstances, the retrograde contamination of the small intestine by bacteria from the hindgut can be an expected occurrence if extremely ground diets are supplied to pigs. Despite the fact that several factors may be involved in the dynamics between host mucosa and non-self antigen interaction, it was hypothesized that the physical form of the diet might have an impact also on Peyer's patches (PPs) morphology (Figure 1), following different potential signs of reaction to the luminal content. The literature reports detailed descriptions of PPs both under physiological (16–18) and pathological conditions (19–23).

As part of a wider research project aiming to explore the effects of the physical form of the diet in pigs, information about the morphology of PPs in relation to different grinding intensities and compaction of the diet turned out to be necessary. Therefore, a feeding trial was undertaken with the purpose of exploring the occurrence of macroscopic and microscopic morphological variations of PPs in the TI of growing pigs, with particular regard to lesions, as a sign of local blockage of potentially harmful microbes.

## Materials and Methods

### Animal and Diet

Animal handling complied with the recommendations of the European Union Directive 2010/63/EU concerning animal care and Consolidated Commission Implemented Decisions 2012/707/EU and 2104/11/EU. The project was approved by the Ethics Committee on Animal Welfare of the Hannover District Government in accordance with German legislation on animal welfare.

A pool of 32 cross-bred weaned pigs (initial BW: 8.30 ± 0.83 kg) out of a total 128 animals, used in different replicates of the same trial, was enrolled in this experiment. All animals were clinically healthy at the start. The dietary treatments used in this study were formulated with identical ingredients and chemical compositions [crude ash, 53 g/kg dry matter (DM); crude protein, 200 g/kg DM; crude fat, 37 g/kg DM; crude fiber, 41 g/kg DM; starch, 470 g/kg DM]. Main ingredients were wheat (39.5%), barley (34%), and soybean meal (20%). A mineral and vitamin premix was added, whereas no organic acids, probiotics, or enzymes were used. Diets were differently processed into four different physical forms to obtain four dietary treatments. The dietary treatments differed in grinding intensities, achieved by variation of mills (roller vs. hammer) and sieves (1 mm vs. 6 mm). Moreover, different compactions of the diet were used. Thus, the differently ground meals underwent pelleting (short conditioning for 15” at a temperature of 67–70°C and pelleting 80–81°C degrees) and extrusion (short conditioning for 15” at 78°C and 115°C temperature). One aliquot of the diet was offered coarsely ground without additional processes and represented the non-compacted diet. To compare the structure of the pelleted, extruded, and unprocessed diets, a wet sieve analysis was performed for all diets (24). For further characterization of the structure of the diet, the discrete mean was calculated according to Fritz et al. (25). The final four dietary treatments differed for the respective granulometry, like that reported in Cappai et al. (15) (Table 1), as follows: finely ground pellet (FP: dMEAN, 0.46 mm); coarsely ground meal (CM: dMEAN, 0.88 mm); coarsely ground pellet (CP: dMEAN, 0.84); and coarsely ground extruded (CE: dMEAN, 0.66). After 1 week of acclimation feeding (phase 1), the four diets were offered for 4 weeks to animals (phase 2), randomly allotted into four experimental groups, according to the dietary treatments. Each group consisted of eight pigs. For each dietary treatment, animals were individually housed without litter in pens with flat concrete floor. All the pigs were fed *ad libitum* (feed supply was adjusted on the basis of maximum capacity of daily DM intake in relation to the animal's BW, determined weekly throughout the trial) and had free access to water. The diet was offered fresh every morning, and the DM content in leftovers was determined daily for every pig. Feed intake was recorded daily and DM intake calculated around the 24 h. Feces were scored though an 8-point scale (1=watery and 8=extremely hard feces), according to Cappai et al. (26). On the last day of the feeding trial, all animals were euthanized 8 h after being given free access to feed.

**Table 1:** Physical forms of the different experimental diets administered to pigs after wet sieving analysis, reporting proportions on DM basis of two sieves with different mesh size (>1 and <0.2 mm) and mean particle size (15).

**Diet**	**I**	**II**	**III**	**IV**
	**Pellet, fine**	**Meal, coarse**	**Pellet, coarse**	**Extruded, coarse**
**Grinding**	**Hammer mill**	**Roller mill**	**Roller mill**	**Roller mill**
>1 mm	8.97	45.8	41.6	29.3
<0.2 mm	42.4	27.2	32.7	43.7
dMean (mm)	0.463	0.880	0.836	0.659

### Sampling at Necropsy and Methods of Evaluation

The protocol consisted of preliminary deep sedation of each pig (intramuscular single injection of Stresnil 40%, corresponding to 2 mg azaperon/kg BW). The intracardiac injection was carried out after 20–25 min from sedation by means of a euthanasic (Tanax® 1 ml/10 kg BW). On an unconscious animal laying on the right side, the needle was inserted between the third and fourth left ribs at the heart level.

The different tracts of the gut were immediately separated with double ties to prevent luminal content from mixing. Subsequently, all tracts were removed carefully from the carcass. Contents of the caudal small intestine and of the cecum were sampled and immediately the pH value was determined. For this purpose, 1 g of chyme was diluted in distilled sterile water added to a volume of 5 ml. The probe of a handy pH-meter was used to record values at a conventional temperature of 25°C. A sample of ileum and cecum chyme from four pigs of each group was analyzed for DM content. In brief, one aliquot of chyme was weighed and overdried at 103.5°C until constant weight.

The last 3 cm of the TI was sampled. Organs were rinsed in water and washed with physiological solution (0.9% NaCl 1N) to allow the gently removal of residual sticking chyme particles from the mucosa. The mucosa of the ileum was inspected for the evaluation of potential lesions in the PPs. Samples were then fixed in buffered formaldehyde (2.5% v/v) for 48 h. Briefly, samples were automatically dehydrated through ascending alcohol series (50° to absolute alcohol), diaphanized in xylene, and embedded in waxy paraffin: conventional histological tissue staining (hematoxylin-eosin, HE) and Mallory-Azan staining were carried out on 2 μm-thick serial sections, obtained at a semiautomatic microtome from the whole length of the PP, throughout the last 3 cm of the TI. On the transversal sections of the whole intestine, the PP appears located in the antimesenterial side of the intestine wall, between ileocecal fold and mesoileum. The macroscopic evaluation pointed to the detection of lesions. The microscopic observations were carried out on a maximum of 10 slides of the small intestine per pig (throughout a sample length of 3 cm), both under light and confocal laser microscope. To allow microscopic analysis, slides were automatically dewaxed in xylene and rehydrated in descending alcohol series (absolute alcohol to 50°), prior to the start of tissue coloration. A binary score (1 = presence vs. 0 = absence) was attributed in case of morphological lesions observed in the PP, as a sign of tissue damage.

### Calculations and Statistical Analysis

Data about animal performance [BW, feed intake, feed conversion ratio (FCR), and fecal score] and presence of lesions in the PP were analyzed through a one-way ANOVA in relation to the dietary group (four treatments). The Tukey test was carried out for grouping analysis. The correlation between the prevalence of microscopic lesions and physicochemical properties of the chyme was carried out through the Pearson's test. All data were analyzed using SAS 9.2 (SAS Inst. Inc. Cary, NC). The statistical significance was set for *p* < 0.05.

## Results

### Overall Assessment of Animals

All the pigs enrolled in the experiment appeared healthy, and no clinical signs of any gastrointestinal dysfunction could be pointed out throughout the trial. Growth performance between groups did not highlight statistically significant differences (Table 2).

**Table 2:** Animal performance in pigs fed with the different diets during the trial.

**Dietary treatments**	**FP**	**CM**	**CP**	**CE**		
- Grinding intensity	Fine	Coarse	Coarse	Coarse		
- Form	Pellet	Meal	Pellet	Extruded		
- Animals, *n*	8	8	8	8	RMSE	*P*–value
**Production performance**						
Daily feed intake (g)	833	1044	856	819	314	0.758
Average daily gain (g)	499	619	497	462	209	0.734
Feed conversion ratio	1.67	1.69	1.72	1.77	0.64	0.758
Fecal score (1–8)	3.58	3.66	3.72	3.68	0.25	0.856
DM content in feces (%)	29.7	27.4	29.2	30.2	0.44	0.001
pH value of feces	6.67	6.81	6.79	6.61	0.09	0.023

Feces appeared normally formed and the fecal score attributed at inspection did not point to differences between groups. Instead, the pH value of feces differed significantly between pigs fed with dietary treatments with the average lowest value in feces of pigs fed with the CE diet. The DM content in feces also differed significantly with the highest content in the feces of pigs fed with the CE diet. No sign of potential gastrointestinal disorders was observed to affect voluntary feed intake or average daily gain of animals throughout the trial. However, the pigs fed with the CE diet displayed a comparatively higher FCR.

The *digesta* of the last part of the small intestine and of the cecum of pigs fed with the four dietary treatments displayed different physicochemical characteristics, as reported in Table 3.

**Table 3:** Physicochemical characteristics of the chyme in the TI and in the cecum, in relation to the dietary treatments.

**Diet**	**FP fine, pellet**	**CM coarse, meal**	**CP coarse, pellet**	**CE coarse, extruded**
Number of animals	8	8	8	8
**pH**				
Terminal *ileum*	6.41 ± 0.31	6.10 ± 0.21	6.52 ± 0.45	5.46 ± 0.20
*Cecum*	5.68 ± 0.23	5.69 ± 0.21	5.73 ± 0.39	5.37 ± 0.24
**DM (g/kg FM)**				
Terminal *ileum*	114 ± 9.02	125 ± 16.6	118 ± 9.12	105 ± 13.4
*Cecum*	108 ± 8.04	137 ± 36.2	125 ± 8.99	113 ± 12.9
**Reaction of the PP**				
Subluminal focal FLN (%)	0.12	0.00	0.00	0.37

### Microscopic Morphology of the PP

**Figure 2:**
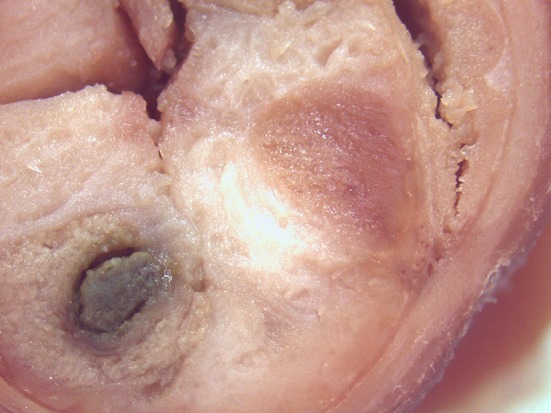
Focal area of degenerated tissue in a PP from a pig fed with the CE diet. Stereomicroscope, hematoxylin-eosin 4×.

**Figure 3:**
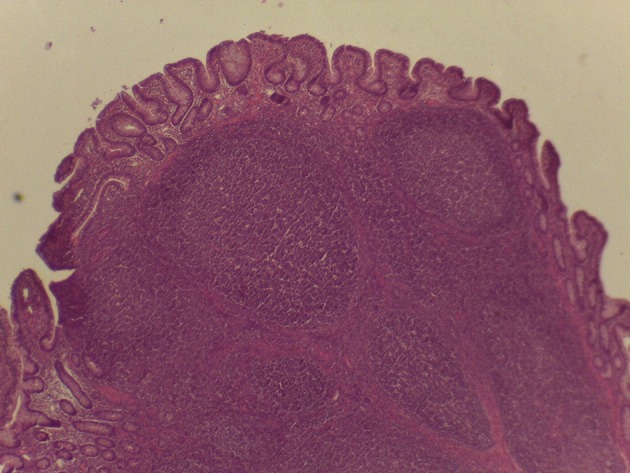
Microscopy of PPs in pigs from the experimental group fed with the FP diet.

**Figure 4:**
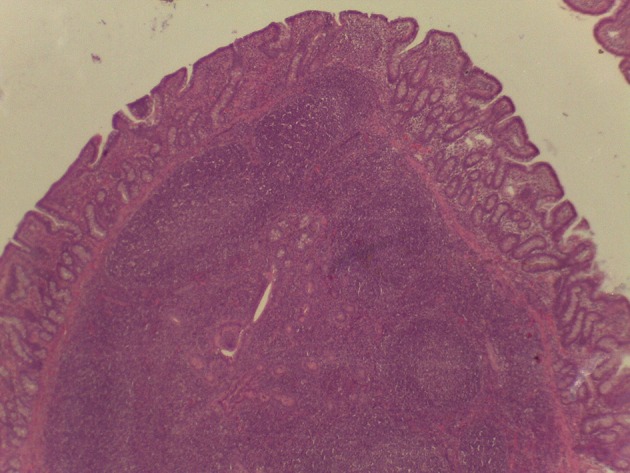
Microscopy of PPs in pigs from the experimental group fed with the CM diet.

**Figure 5:**
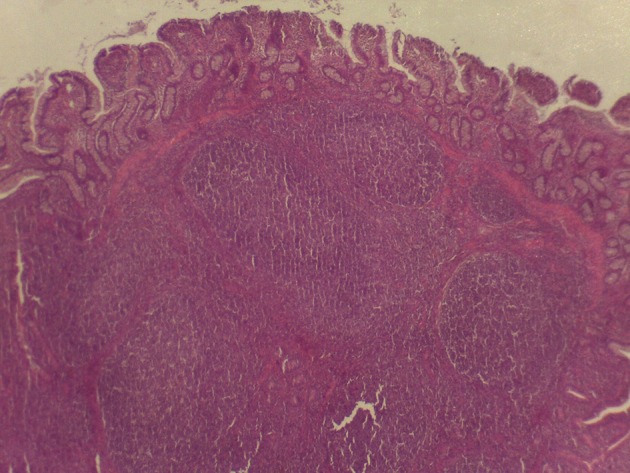
Microscopy of PPs in pigs from the experimental group fed with the CP diet.

**Figure 6:**
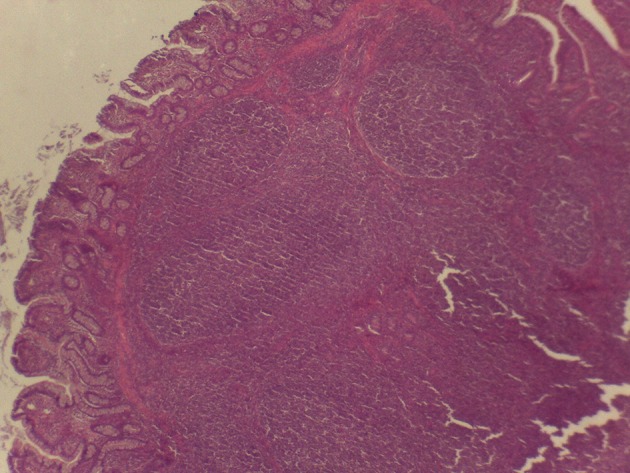
Microscopy of PPs in pigs from the experimental group fed with the CE diet.

**Figure 7:**
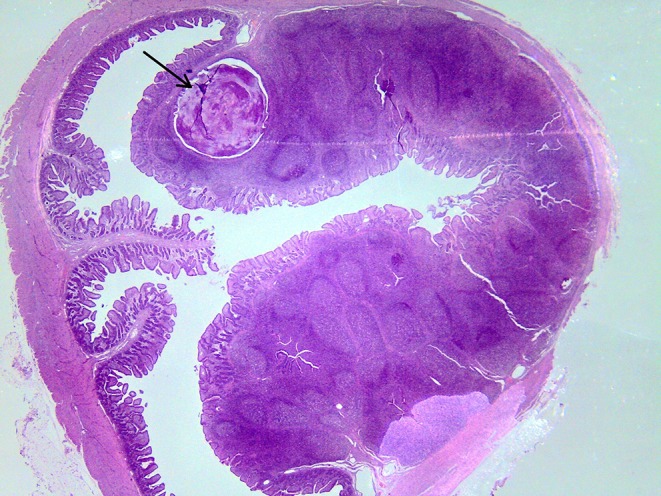
Subluminal lesion (arrow) characterized by liquefactive necrosis of tissue in the PP of a pig fed with the FP diet.

On macroscopic and microscopic scoring of PPs based on checking lesions (0 = absence vs.1 = presence), the presence of subluminal focal liquefactive necrosis mirrored local reaction leading to tissue damage (Figure 2). This finding was recorded once in the last 3 cm of the TI in three pigs out of eight fed with the CE diet and in one pig out of eight fed with the FP diet. No other focal necrotic areas were found in pigs fed with the CM and CP diets. Despite the presence of subluminal degeneration (Figure 2), which was found solely in pigs fed with the FP and CE diets (which were also the diets with the smallest average particle size), no significant difference was found between groups (Figures 3–6). The prevalence of the FLN (Figure 7) negatively and significantly correlated with the pH value of the chyme of the small intestine (ρ = −0.361; *p* = 0.026). In support to the significant difference of the pH values recorded in the small intestine content among groups, it is also pointed out that the DM content and pH of feces differed significantly between groups.

## Discussion

The present trial aimed to shed a new light on the potential effects of the physical form of the diet on the morphology of the PP in the TI of the growing pig. The rationale behind this investigation moved from the results obtained in previous trials, which highlighted different morphometry and functional efficiency of the IV in relation to the physical form of the diet (15). Such different morphology of the IV was interpreted in light of concomitant changes in the physical and chemical properties of the chyme and the composition of microbial populations of *digesta* in the small and large intestines, as a result of different physical forms of one same diet (8). If ascendant microbial colonization from the hindgut occurs, then PPs in the TI acts as a barrier of immune local defense. The evaluation of local response of PP in conventionally raised pigs is an extremely difficult task; however, the presence of subluminal focal lesions may be considered as a sign of reaction against pathogens. It is here to point out that one first limit of this investigation is that the focus on the PP restricted to the last 3 cm of the TI is not representative of the local immune response of the whole gut, but, as a region of interest, would probably serve to explore the involvement of PP in the case of a less efficient backflow prevention. In fact, the FP and CE turned out to point to undesirable outcomes of chyme in previous trials (8, 15). This being said, dietary tactics for predisposing to optimal acquisition of immunological competence in the young animals at farm level involve aspects related to both health and welfare. In the man and the pig, the ileum ends into a developed IV into the cecum. The role of the IV seems to be pivotal in limiting the pathogen backflow from the hindgut (15, 27). It is assumed that coliforms largely hosted in the large intestine can increase in the chyme of the ileum (CFU/g of *digesta*) when finely ground and markedly treated (pelleted and extruded) diets were offered to pigs (8). In view of such findings, it was supposed that the physical form of the diet could play an indirect role in determining different antigenic potentials of the luminal content in the TI, indirectly leading to different extents of PP antigenic exposure to luminal content. Negative findings of subluminal focal lesions in PP of piglets fed with the CM and CP dietary treatments do not obviously exclude lesions in the PPs of the small intestine uphill (before the last 3 cm of TI considered here). It could be argued that the lesions observed in PPs may happen more frequently in piglets fed with the FP and CE dietary treatments, which, however, do not show any apparent digestive dysfunction nor differences in FCR. Whether different subtypes of inflammatory cells were involved in the immune reactions was not investigated further in this trial, and this is another limit of our investigation so far. Nevertheless, evidence from this first description can reasonably pave the way to further exploration. In light of what was pointed out in this feeding trial, it appears absolutely necessary to carry out cytological analysis of PP that may reveal the immune responsiveness on the basis of activation of the different immune cell clusters. The role of the physical form of the diet in predisposing to different antigenic potentials of the chyme of the small intestine and the consequent exposure of PPs should not be underestimated. In fact, significant considerations can be drawn out of the results obtained in this trial, in light of what was observed in previous experiments by the same group of researchers (8, 15). Results obtained from the experimental feeding seem to suggest that the physical form of the diet could be considered as a triggering factor for PP responsiveness, where physicochemical properties of the chyme can lead to different mechanic consequences, thus favoring the retrograde colonization of the small intestine by hindgut bacteria. In the literature, part of intestinal diseases caused by pathogenic bacteria was associated with dietary cofactors (28). In fact, it is known that microbial populations of the lower tracts of the intestine share molecular patterns, which are used by the host for innate immune response (toll-like receptors, TLRs) (20, 29). With particular regard to PP histoarchitecture, the follicle associated epithelium (FAE) possesses modified enterocytes, named M cells (30). Such highly specialized cells of the mucosal layer are enterocytes that switched to take part in the immune function (30). This marked plasticity of FAE is due to the key role played by M cells. M cells are specialized in the transcytosis of intact luminal materials like soluble proteins, antigens, bacteria, and viruses (31). That way, the luminal antigen is carried by M cells to the immune cells of the sub-epithelial dome (SED), represented by macrophages, B cells, T cells, and dendritic cells (DCs). In addition, the literature (32) points to alternative ways for pathogenic bacteria to gain extra-enteral tissues. DCs are capable of penetrating the monolayer mucosa of the gut (FAE) to capture pathogenic bacteria from the lumen into the PP. The coarsely ground meal diet led to comparatively lower content of DM in feces and higher pH values. This is probably due to the fact that the passage rate in the large intestine is enforced when a coarsely ground diet is administered to pigs, thus inducing the animal to shed feces with higher water content (and increase bacterial clearance). Consequently, pH values turned out to be comparatively higher when the CM diet was offered, likely due to a reduced transit time of the chyme in the large intestine. Indeed, the significantly negative correlation between the pH value of the chyme from the last portion of the small intestine and the prevalence of FLN in the PP may represent different aspects of the whole. It is worth noting that, in light of the results obtained in this trial, Hyland et al. (33) reported that *Salmonella* strains with invasion-associated genes may not induce effective innate or adaptive response, unless a massive infection occurs. In fact, the same authors reported that massive infections of *Salmonella* with invasion-associated genes are capable of stimulating the interleukin (IL-1β and IL-8) expression for immune cell activation. Thus, continuous and subliminal stimulation from hindgut microbes with invasive traits like some *Salmonella* strains may not aid to develop the immune competence of the host, but may rather represent a continuous and repeated local challenge, with potential negative underlying effects on animal health and welfare. In view of the findings observed in this trial, prophylactic strategies to prevent the spread of pathogens should be supported by the adequate physical form of the diet administered to young piglets at the farm level.

## Conclusion

Marked grinding intensities and compaction processes of one same diet might be accounted as cofactors contributing to the different physicochemical characteristics of the *digesta* in the small intestine. Local response to gut content was observed in the PPs in the TI of experimental pigs fed with different dietary treatments. Despite the fact that the histological examination of this very restricted site is not representative of the complexity of gut immune response, our findings point to the presence of subluminal focal liquefactive necrosis in PP of the TI as a region of interest, in particular when extremely ground and compacted diets are offered to growing pigs. Despite the fact that the differences in PP morphology and lesion presence following the dietary treatments were not significant between animals of different groups, this fact should not lead to underestimate the subclinical repeated antigenic stimulation. Production performance of animals was not affected by the different physical forms of the same diet during the period limited to this investigation, whereas differences were detected with regard to the DM content and the pH value of the feces of pigs fed with different dietary treatments.

## Data Availability Statement

The datasets generated for this study are available on request to the corresponding author.

## Ethics Statement

The project was approved by the Ethics Committee on Animal Welfare of the Hannover District Government in accordance with German legislation on animal welfare.

## Author Contributions

MC, MA, and SS participated in the design of the study, carried the feeding trial, monitored animal performance, and did histological investigations. MC and CD did calculations and statistical analysis. MC and WP drafted the paper. JK conceived the study, participated in its design and coordination, and helped to draft the manuscript. All authors read and approved the final manuscript.

## Conflict of Interest

The authors declare that the research was conducted in the absence of any commercial or financial relationships that could be construed as a potential conflict of interest.
